# First person – Juan Garrido-Maraver

**DOI:** 10.1242/bio.050112

**Published:** 2020-01-14

## Abstract

First Person is a series of interviews with the first authors of a selection of papers published in Biology Open, helping early-career researchers promote themselves alongside their papers. Juan Garrido-Maraver is first author on ‘Forcing contacts between mitochondria and the endoplasmic reticulum extends lifespan in a *Drosophila* model of Alzheimer's disease’, published in BiO. Juan conducted the research described in this article while a postdoctoral scientist in L. Miguel Martins's lab at the MRC Toxicology Unit, University of Cambridge, Leicester, UK. He is now a postdoctoral scientist in the lab of Álvaro A. Tavares at the Centre for Biomedical Research (CBMR), University of Algarve, Faro, Portugal, investigating molecular mechanisms linked to human diseases from a therapeutic point of view.


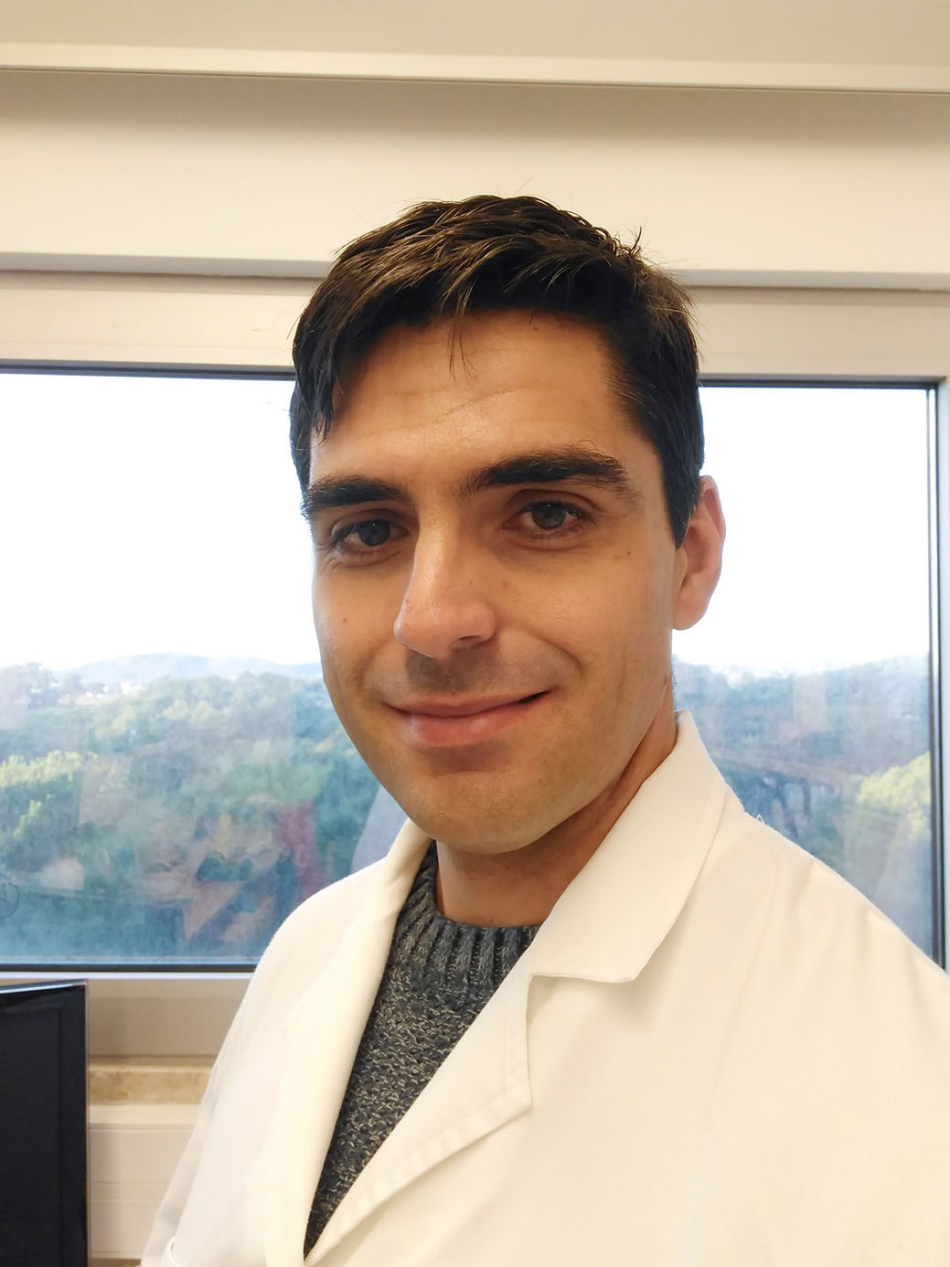


**Juan Garrido-Maraver**

**What is your scientific background and the general focus of your lab?**

My scientific career started when I joined the Andalusian Centre for Developmental Biology (CABD) in Seville (Spain). There I conducted my PhD in Dr José Antonio Sánchez-Alcázar′s group investigating MELAS syndrome, a disease caused by defects in mitochondria. My research focused on how cells derived from patients can manifest different pathophysiology depending on the mutational load, AMP-activated protein kinase (AMPK) sensitivity, and the balance between mitochondrial biogenesis and clearance. In parallel, I also worked under the close supervision of Professor Acaimo González-Reyes who showed me the power of fruit flies, *Drosophila melanogaster*, as a model organism. In his lab I investigated the microtubule network rearrangements during apoptosis in *Drosophila* oogenesis. After finishing my PhD programme, I did my first postdoc in Dr L. Miguel Martins's group at the MRC Toxicology Unit in Leicester. The aim of Miguel Martins's research programme is to understand the cellular and molecular mechanism that mitochondria use to cope with toxic insults. There, I combined my previous experience on mitochondria and *Drosophila* to study the interface between mitochondria and the endoplasmic reticulum in models of neurodegenerative diseases.

**How would you explain the main findings of your paper to non-scientific family and friends?**

Mitochondria and the endoplasmic reticulum are two cellular components with specialized functions in eukaryotes. Whereas the endoplasmic reticulum plays a major role in storing calcium, processing proteins for modification and delivery to other organelles, mitochondria are responsible for the conversion of energy stored in the chemical bonds of nutrients into ATP, the cellular energy currency. Besides their own functions, both mitochondria and endoplasmic reticulum come into proximity to form contact sites where they exchange molecules like calcium. These contacts are dynamic and respond to the metabolic state of cells. The disturbances in the interaction between both organelles have been linked to neurodegeneration.

Most of the investigations in the field study the interaction between mitochondria and endoplasmic reticulum at a cellular level. We wondered about the physiological consequences of enhancing these contacts in animals. The fruit fly, *D. melanogaster*, is per excellence one of the most used model organisms to investigate human diseases. We used a genetic approach to explore the consequences of enhancing the contacts between mitochondria and endoplasmic reticulum. We expressed a synthetic linker that tethers both organelles in flies. We showed that forcing these contacts increases mitochondrial calcium uptake, accompanied with an increase of oxidative stress that ultimately improves locomotion and extends lifespan of flies. In neurodegenerative diseases, we studied a *Drosophila* model of Alzheimer's disease based in the overexpression of toxic amyloid beta. The accumulation of toxic amyloid beta in neurons causes Alzheimer's disease. Our results show that the linker expression improves motor impairment and extends lifespan of flies modelling Alzheimer's.

**What has surprised you the most while conducting your research?**

In the last decade, the study of contacts between mitochondria and endoplasmic reticulum has emerged strongly and the knowledge on this field is developing at a very fast pace. Despite that, there is not a clear consensus about the functional role of these contacts. We accepted the challenge and explored the consequences of manipulating these contacts in animals. One of the most surprising aspects conducting my research was to observe that forcing contacts does not imply a negative outcome for fruit flies, quite the opposite, it improves locomotion and lifespan. Somehow, the increase of the interaction between these two organelles make a substantial and beneficial response in the behaviour of animals. The fact that forcing contacts promotes locomotion and extends lifespan of fruit flies uncovers the therapeutic potential that its manipulation may have.

**Figure:**
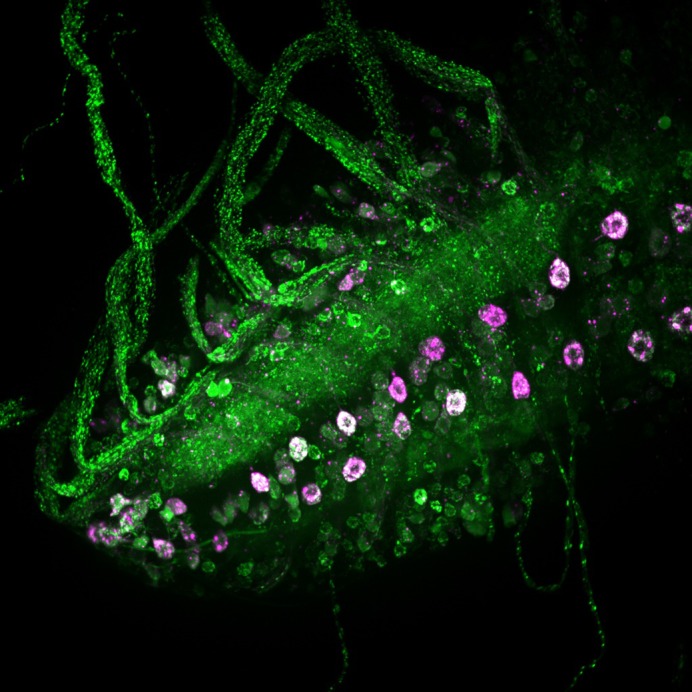
**Dopaminergic neurons in third instar larvae brains co-expressing a synthetic linker that tethers mitochondria and endoplasmic reticulum (magenta) and a mitochondrial calcium reporter (green).**

“[…] forcing contacts does not imply a negative outcome for fruit flies, quite the opposite, it improves locomotion and lifespan.”

**What are the potential implications of these results for your field of research?**

Providing evidence that tethering two organelles improves the physiological behaviour of animals is interesting from a biological point of view. More interestingly, the fact that the linker improved the phenotype of a *Drosophila* model for Alzheimer's disease suggests that the increase of these contacts might have a therapeutic potential in neurodegeneration. Considering these results, a new door is opening to study the effect of pharmacological compounds on contacts in models for human diseases where interactions between these organelles are impaired. Even though more investigations are needed, the manipulation of these contacts might be considered as a promising strategy for applications in disorders with physical locomotor disabilities.

**What changes do you think could improve the professional lives of early-career scientists?**

I think that the funding opportunities need improvements. The traditional model of hiring postdocs forces young scientists to jump from one lab to the next until, if lucky, they get a more stable position. Sadly, the end of the contract normally marks a deadline for getting their papers out that conflicts with deepening work into the projects. The current model requires a fundamental change to move toward longer contracts allowing young scientists to make the best of their projects with a fair work/life balance. Otherwise, we become simple and temporary tools to address projects that normally end with their contracts. On the other hand, I believe that the successful career of young scientists also depends on the support of their mentors and institutions. Making correct decisions and building a career up truly needs the collaboration of the surrounding experienced scientists. I believe the collaboration of career advisors and senior scientists in the institutions is crucial to provide the guidance required in order for young scientists to launch their professional careers.

**What's next for you?**

After finishing my contract in the UK, I moved to Faro to start my second postdoc. I joined Álvaro Tavares's lab at Centre for Biomedical Research (CBMR) where I am addressing a new and exciting project on chromosomal segregation using *Drosophila* as a model organism. Here I am exploring the regulatory components of the mitotic machinery and the molecular mechanisms by which chromosome mis-segregation can lead to cancer. I love the challenge of exploring the pathological mechanisms of human diseases and with this new project I continue enriching my skills and knowledge. The idea of shedding light on the molecular mechanisms underlying cancer encourages me to give the best of myself to do science.
